# A genomic approach to bacterial taxonomy: an examination and proposed reclassification of species within the genus *Neisseria*

**DOI:** 10.1099/mic.0.056077-0

**Published:** 2012-06

**Authors:** Julia S. Bennett, Keith A. Jolley, Sarah G. Earle, Craig Corton, Stephen D. Bentley, Julian Parkhill, Martin C. J. Maiden

**Affiliations:** 1 Department of Zoology, University of Oxford, Oxford OX1 3PS, UK; 2 The Wellcome Trust Sanger Institute, Wellcome Trust Genome Campus, Hinxton CB10 1SA, UK

## Abstract

In common with other bacterial taxa, members of the genus *Neisseria* are classified using a range of phenotypic and biochemical approaches, which are not entirely satisfactory in assigning isolates to species groups. Recently, there has been increasing interest in using nucleotide sequences for bacterial typing and taxonomy, but to date, no broadly accepted alternative to conventional methods is available. Here, the taxonomic relationships of 55 representative members of the genus *Neisseria* have been analysed using whole-genome sequence data. As genetic material belonging to the accessory genome is widely shared among different taxa but not present in all isolates, this analysis indexed nucleotide sequence variation within sets of genes, specifically protein-coding genes that were present and directly comparable in all isolates. Variation in these genes identified seven species groups, which were robust to the choice of genes and phylogenetic clustering methods used. The groupings were largely, but not completely, congruent with current species designations, with some minor changes in nomenclature and the reassignment of a few isolates necessary. In particular, these data showed that isolates classified as *Neisseria polysaccharea* are polyphyletic and probably include more than one taxonomically distinct organism. The seven groups could be reliably and rapidly generated with sequence variation within the 53 ribosomal protein subunit (*rps*) genes, further demonstrating that ribosomal multilocus sequence typing (rMLST) is a practicable and powerful means of characterizing bacteria at all levels, from domain to strain.

## Introduction

The genus *Neisseria* comprises Gram-negative oxidase-positive diplococci, many of which are harmless commensal inhabitants of the mucosal and dental surfaces of humans (Zaura *et al.*, 2009). The genus contains two human pathogens that cause very different diseases, both of global significance: *Neisseria meningitidis*, the meningococcus, which causes meningitis and septicaemia; and *Neisseria gonorrhoeae*, the gonococcus, which causes gonorrhoea and, occasionally, disseminated infections. Conventionally, species of the genus *Neisseria* are distinguished based on their phenotypic properties, using assays such as carbohydrate utilization and enzyme substrate tests. While these techniques are generally satisfactory for the identification of the meningococcus, gonococcus and the lactose-fermenting organism *Neisseria lactamica*, misclassification is not uncommon using these methods and can have important medical consequences (Dossett *et al.*, 1985).

A number of approaches have been used to explore the relationships and species assignment of the genus *Neisseria*, including DNA–DNA hybridization (Tønjum *et al.*, 1989), numerical taxonomy (Barrett & Sneath, 1994), 16S rRNA gene sequencing (Harmsen *et al.*, 2001) and analysis of the seven housekeeping gene fragments (Bennett *et al.*, 2007) used in MLST (Maiden *et al.*, 1998). DNA–DNA relatedness studies have shown that four members of the genus, *N. meningitidis*, *N. gonorrhoeae*, *N. lactamica* and *Neisseria polysaccharea*, are closely related (Guibourdenche *et al.*, 1986), although two cause distinct human diseases. Phylogenies constructed from 16S rRNA gene sequences provide sufficient resolution to distinguish the genus *Neisseria* from its close relatives; however, *Neisseria* isolates classified as distinct species may have identical or very similar 16S rRNA gene sequences to other species within the genus (Harmsen *et al.*, 2001).

The genus *Neisseria* is an instructive model system for examining the relationships of epidemiology, population genetics and evolution with the emergence of distinct phenotypes, especially those associated with invasive disease (Maiden, 2008). Members of the genus are naturally competent for the uptake of DNA by transformation, which is mediated by a specific uptake mechanism involving DNA uptake sequences (DUS) (Treangen *et al.*, 2008). For over 20 years, the genus has played a central part in establishing the importance of horizontal genetic exchange in bacterial population structure and evolution (Maynard Smith *et al.*, 1991; Spratt, 1988). Genomic studies of individual isolates have been combined with population analyses using MLST data (Bennett *et al.*, 2010). These studies suggest first that the accessory genome, which includes genes thought to be associated with the ability to cause invasive disease, is widely shared among pathogenic and non-pathogenic members of the genus (Marri *et al.*, 2010), and second, that sequence polymorphism in core genes, those present in all isolates, is important in defining the groups of genetically related isolates currently assigned species status (Bennett *et al.*, 2010).

The present study analysed *Neisseria* species described in Bergey’s Manual of Systematic Bacteriology (Tønjum, 2005) to determine the phylogenetic relationships among these species and specifically their relationship to *N. meningitidis*. Species structure within the genus was investigated using whole-genome sequence data from 15 *Neisseria* species: *N. meningitidis*, *N. gonorrhoeae*, *N. lactamica*, *N. polysaccharea*, *Neisseria cinerea*, *Neisseria flavescens*, the *Neisseria subflava* biovars *Neisseria subflava*, *Neisseria perflava* and *Neisseria flava*, *Neisseria mucosa* and the *Neisseria mucosa* variant *Neisseria mucosa* var. *heidelbergensis*, *Neisseria sicca*, *Neisseria elongata* subsp. *glycolytica*, *Neisseria bacilliformis*, *Neisseria macacae*, *Neisseria canis*, *Neisseria dentiae* and *Neisseria weaveri.* Type strains from 12 of the commensal *Neisseria* were included as reference species (see Table S1 available with the online version of this paper). The type strains of *N. meningitidis*, *N. gonorrhoeae* and *N. lactamica* were not included, as fully annotated genomes were already available for these species and their species status is not in doubt.

The database platform Bacterial Isolate Genome Sequence Database (BIGSdb) (Jolley & Maiden, 2010), which is able to store genomic sequence data and has the capacity to define and identify any number of loci and genetic variants at these loci, was employed to identify nucleotide variation in genes present in all taxa. A reference gene approach using previously annotated *Neisseria* genomes for initial locus designation (Bennett *et al.*, 2010) identified successive sets of genes that generated distinct groups of isolates, with the set of 53 ribosomal protein subunit (*rps*) genes, used in the ribosomal MLST (rMLST) typing scheme (Jolley *et al.*, 2012), providing a minimal set of genes that clustered the isolates into groups, broadly consistent with current species assignments. These data demonstrate that some isolates currently in culture collections have been misnamed and that some minor changes in nomenclature are required.

## Methods

### 

#### Isolates.

A total of 36 *Neisseria* isolates were sequenced *de novo*, four *N. lactamica* isolates obtained from asymptomatic carriage in children in Oxfordshire (Bennett *et al.*, 2005) and 32 isolates from the Culture Collection of the University of Göteborg (CCUG), Sweden (Tables S1 and S2). The CCUG isolates comprised 28 isolates designated human commensal *Neisseria*: five *N. polysaccharea*, four *N. cinerea*, three *N. flavescens*, one *N. mucosa*, one *N. mucosa* var. *heidelbergensis*, three *N. sicca*, one *N. bacilliformis* and 10 *N. subflava*, which comprised the biovars *N. perflava* (three), *N. subflava* (five) and *N. flava* (two). In addition, the CCUG isolates included four *Neisseria* (*N. canis*, *N. dentiae*, *N. weaveri* and *N. macacae*) not isolated from humans.

#### Microbiology and sequencing.

Freeze-dried bacterial isolates were inoculated onto Columbia horse-blood agar (Oxoid) and incubated for 24 h at 37 °C in a 5 % CO_2_ atmosphere. Genomic DNA was prepared using the Wizard Genomic DNA Purification kit (Promega), according to the manufacturer’s instructions. Standard Illumina multiplex libraries were generated according to the manufacturer’s instructions, using 1 µg genomic DNA sheared to between 200 and 300 bp using a Covaris E210 acoustic shearing device. Up to 12 libraries were pooled together in an equimolar ratio for sequencing in one flow cell lane on the Illumina Genome Analyzer II platform; 54 bp paired end reads were generated. Genomes were assembled using Velvet 1.0.10 (Zerbino & Birney, 2008); the assembly process was optimized using default parameters for the VelvetOptimizer script provided with the Velvet software package. Assembly data are available as Table S2.

#### Public sequence data.

Whole-genome data from 19 isolates were downloaded from either the Integrated Microbial Genomes (IMG) database found at http://img.jgi.doe.gov/cgi-bin/w/main.cgi (Markowitz *et al.*, 2010) or GenBank (http://www.ncbi.nlm.nih.gov/genbank/). These data included genome sequences of five *N. meningitidis* isolates (Bentley *et al.*, 2007; Parkhill *et al.*, 2000; Peng *et al.*, 2008; Schoen *et al.*, 2008; Tettelin *et al.*, 2000), six *N. gonorrhoeae*, including one published genome (Chung *et al.*, 2008), one *N. lactamica* (Bennett *et al.*, 2010), and one each of *N. cinerea*, *N. flavescens*, *N. mucosa*, *N. sicca*, *N. polysaccharea*, *N. subflava* and *N. elongata* subsp. *glycolytica* (Marri *et al.*, 2010) (Table S1).

#### Uploading and annotation of sequence data with BIGSdb.

All genome data were uploaded to BIGSdb, along with available taxonomic and provenance data and links to the appropriate PubMed record; these data are accessible through the PubMLST database (http://pubmlst.org). The identifiers used for the isolates were usually those provided with the isolates, but all other known names associated with these isolates were included as aliases. Where isolates were obtained from culture collections, the culture collection name was accorded priority and the species designation provided with the isolate was used. Genes within the sequences were annotated with the tagging functionality included in BIGSdb (Jolley & Maiden, 2010; Jolley *et al.*, 2012). Briefly, known genes were used as query strings for iterative searches with progressively decreasing stringency of the whole-genome data by means of the blastn and tblastx algorithms (Altschul *et al.*, 1997). This process identified likely genes, which were tagged in the database, enabling them to be extracted and exported in formats suitable for various analyses. For a given locus, each unique complete sequence identified was assigned an arbitrary allele number. Allele sequences were manually checked to ensure that only in-frame sequences without internal stop codons were included and that the sequences began at common start codons where possible. For a small number of the gene sequences analysed, some of the data were missing from the ends of the contigs assembled from the short-read data, and in a few cases, apparent frameshift mutations were present, resulting in internal stop codons. These data were included in the analysis but were not assigned allele designations. Gene sequences from the isolate database were exported as XMFA files containing each locus as an aligned block, and then converted to fasta format for importing into mega version 5.0 (Tamura *et al.*, 2007).

#### Analyses.

The BIGSdb genome comparator tool, which identifies loci shared among genomes and their allelic diversity, was used to detect genes present among all taxa. The annotated gene sequences from the published FAM18 genome (Bentley *et al.*, 2007) were compared to whole-genome sequence data from 54 isolates using the following parameters: minimum percentage identity of 50, minimum percentage alignment of 30 and blastn word size of 11. As the search used nucleotide sequences, it would be expected to retrieve only conserved protein-coding genes. This level of stringency was chosen to ensure that only homologous genes were analysed.

Neighbor-joining phylogenies (Saitou & Nei, 1987) and a neighbor-net phylogeny (Bryant & Moulton, 2004) using nucleotide p-distances were constructed in mega version 5.0 and SplitsTree version 4 (Huson & Bryant, 2006), respectively. Genetic distances were calculated using mega version 4.0, DnaSP version 5 (Librado & Rozas, 2009) was used to calculate shared polymorphisms and fixed differences, and Arlequin version 3.11 (Excoffier *et al.*, 2005) was used to calculate fixation index (*F_ST_*) values.

## Results and Discussion

While bacterial nomenclature is covered by the International Code of Nomenclature of Bacteria (Lapage *et al.*, 1992), the bacterial species concept remains contentious at both a conceptual (Doolittle, 2008) and practical level (Stackebrandt *et al.*, 2002). As increasing volumes of nucleotide sequence data become available from across the bacterial domain, the need for the systematic organization of bacterial groups becomes increasingly important (Achtman & Wagner, 2008). The long-established gold standard of DNA–DNA relatedness (Wayne *et al.*, 1987) is not easily applied to all specimens and cannot resolve closely related members of certain groups, even though these may have distinct phenotypic properties deserving of distinct species status (Achtman & Wagner, 2008). There is general agreement that taxonomic schemes should be backwards-compatible, phylogenetically consistent and reflect genetic relatedness (Stackebrandt *et al.*, 2002; Wayne *et al.*, 1987); however, there is no consensus as to how this is best achieved (Achtman & Wagner, 2008). Approaches based on sequencing of multiple chromosomal loci, first envisioned in the late 1980s (Wayne *et al.*, 1987), have been proposed (Gevers *et al.*, 2005), but no practical method is yet in universal use. Here, we explore the use of genomic sequence data from members of the genus *Neisseria* to define species groups, concentrating on sequence variation in comparable subsets of genes present among all isolates examined.

### 16S rRNA and MLST gene phylogenies

**Fig. 1.**
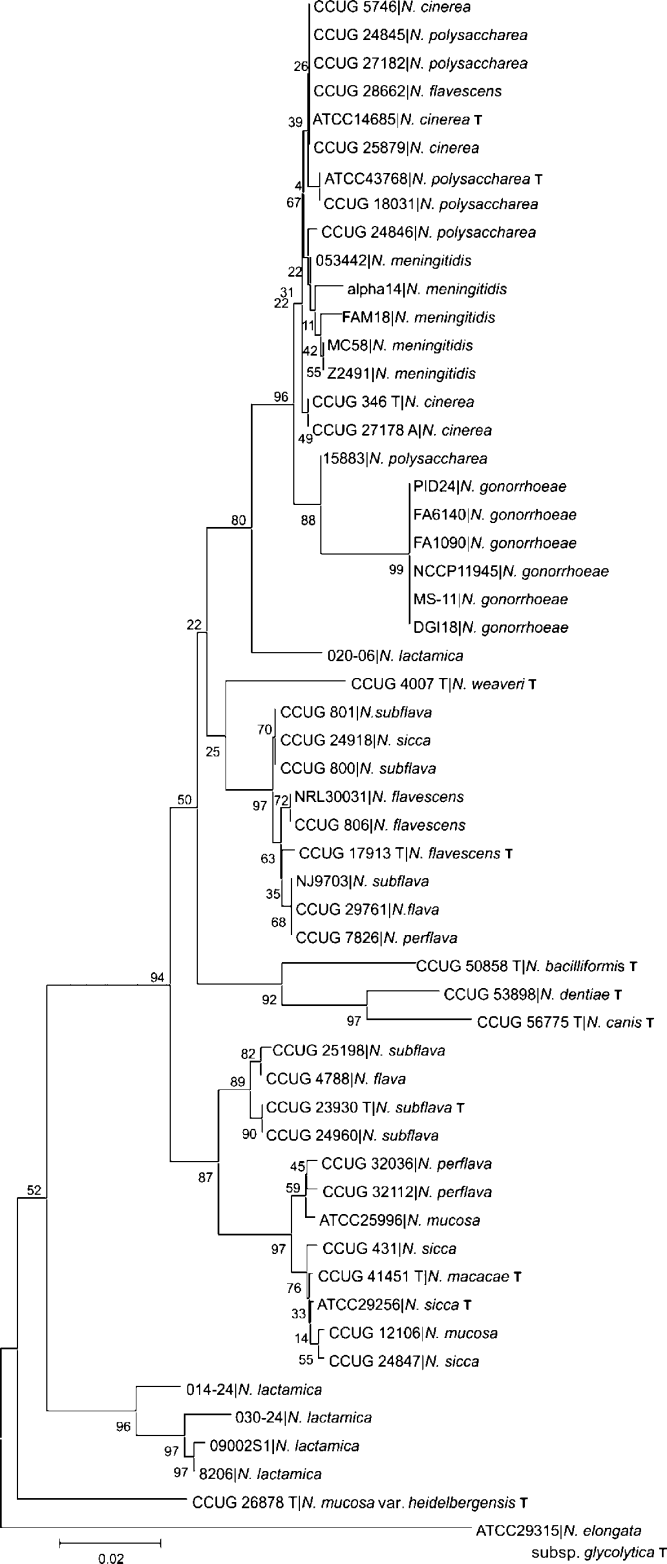
Evolutionary relationships among *Neisseria* based on 16S rRNA fragments. The evolutionary history was inferred using the neighbor-joining method. The percentage of replicate trees in which the associated taxa clustered together in the bootstrap test (500 replicates) is shown next to the branches. The analysis involved 55 nt sequences consisting of 456 nt. ‘T’ denotes type strain.

A 456 bp gene fragment was extracted from one 16S rRNA gene from each of the 55 genomes examined, resulting in 36 unique alleles with an overall mean p-distance among alleles of 0.053. A neighbor-joining phylogeny generated with these data was poorly congruent with species designations of the isolates and only one group contained isolates assigned to a single species (*N. gonorrhoeae*) (Fig. 1). Consistent with previous findings (Tønjum, 2005), some isolates assigned the same species names occupied very different positions in the tree. For example, while four *N. lactamica* sequences formed a distinct group, the 16S rRNA sequence from *N. lactamica* isolate 020-06 was highly divergent. Furthermore, one cluster included species described as *N. meningitidis*, *N. polysaccharea*, *N. cinerea* and *N. flavescens*, and isolates thought to be *N. polysaccharea* and *N. flavescens* had 16S rRNA gene sequences identical to the type strain of *N. cinerea* (ATCC 14685). Other strains described as particular species did not cluster with the type strains of their designated species, indicating that further taxonomic investigation is required to clarify the species identity of these strains. These data confirmed that the 16S phylogeny was not useful for species assignment within the genus, due to a combination of low and unevenly distributed sequence diversity – a consequence of shared ancestry, inter-species horizontal genetic exchange (Smith *et al.*, 1999) or both. The 16S rRNA phylogeny was not used further in this analysis.

Gene fragments corresponding to the loci used for MLST were extracted from the database, concatenated and used to generate a neighbor-joining phylogeny, effectively the multilocus sequence analysis (MLSA) approach (Gevers *et al.*, 2005). This phylogeny (Fig. S1), generated groups that were consistent with microbiological designations for isolates characterized as *N. meningitidis*, *N. gonorrhoeae* and *N. lactamica*, as has been described previously (Bennett *et al.*, 2007). All of the isolates microbiologically assigned to *N. cinerea* clustered with the type strain of *N. cinerea* (ATCC 14685), along with one isolate previously identified as *N. flavescens* (CCUG 28662). The phylogeny indicated that this isolate could be a misidentified *N. cinerea*. Three *N. polysaccharea* isolates (CCUG 24845, CCUG 24846 and CCUG 18031) grouped with the *N. polysaccharea* type strain ATCC 43768 (Riou *et al.*, 1983), but *N. polysaccharea* isolates 15883 and CCUG 27182 did not, with 15883 more distantly related. The other isolates did not cluster clearly into species-specific groups, indicating that variation at the MLST loci provides insufficient power to resolve all *Neisseria* into distinct species groups.

### Examination of common genes sets

**Fig. 2.**
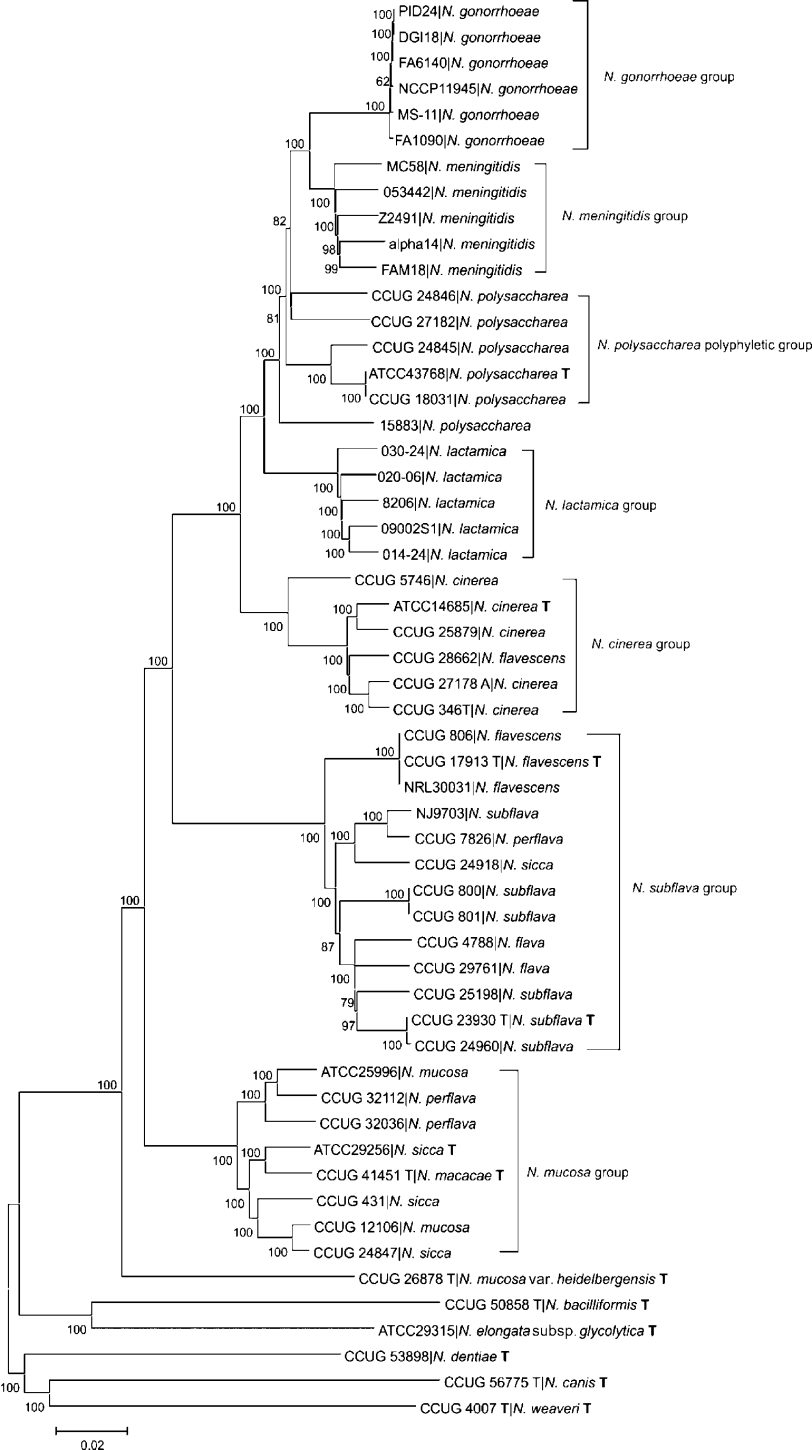
Evolutionary relationships among *Neisseria* based on concatenated sequences from 246 genes. The evolutionary history was inferred using the neighbor-joining method. The percentages of replicate trees in which the associated taxa clustered together in the bootstrap test (500 replicates) are shown next to the branches. The analysis involved 55 nt sequences consisting of 190 534 nt. ‘T’ denotes type strain.

The genome comparator module of BIGSdb was employed to identify comparable coding sequences shared among the *Neisseria* genomes, with *N. meningitidis* FAM18 used as the reference genome. Using blastn, 246 genes, totalling 190 534 nt and amounting to 8.68 % of the query genome, were identified in all genomes (Table S3) using the blastn criteria described. A neighbor-joining phylogeny reconstructed from concatenated sequences of these genes generated seven groups (Fig. 2). Four of the groups comprised isolates belonging to single species: *N. meningitidis*, *N. gonorrhoeae*, *N. lactamica* and *N. cinerea*, with *N. flavescens* CCUG 28662 grouped again with *N. cinerea*, confirming the suggestion from MLST data that this isolate was a misidentified *N. cinerea*.

A further group, which contained the *N. subflava* type strain (CCUG 23930 T), consisted mainly of species defined in Bergey’s Manual of Systematic Bacteriology (Tønjum, 2005) as *N. subflava* and *N. subflava* biovars. There was little distinction among the biovars *N. subflava* biovar *subflava*, *N. subflava* biovar *perflava* and *N. subflava* biovar *flava*, confirming that they are variants of the same species. The three *N. flavescens* isolates were also included in this group, and were almost identical, consistent with their evolution from a single clone (Branham, 1930). The similarity between *N. flavescens* and the *N. subflava* biovars suggests that this species may also require reclassification as an *N. subflava* biovar. The *N. sicca* isolate (CCUG 24918) which clustered in this group is likely to be a misidentified *N. subflava* species.

The sixth group, distinct from the other five, consisted of isolates described as the following species: *N. mucosa*, *N. sicca*, *N. perflava* and the non-human isolate *N. macacae*. Here, the term ‘*N. mucosa* group’ has been used to define these organisms, as *N. mucosa* (originally given the name *Diplococcus mucosus* by Von Lingelsheim in 1906) was the first of these species to be identified (Tønjum, 2005). *N. mucosa* var. *heidelbergensis* (Berger, 1971) was shown to be distinct from the other *N. mucosa* isolates, as described by Tønjum (2005). Phenotypically and biochemically, *N. sicca* and *N. mucosa* are very similar, except that *N. mucosa* reduces nitrates and forms mucoid colonies, whereas *N. sicca* does not and forms dry, wrinkled colonies (Tønjum, 2005). This, taken together with the genetic data, suggests that these two species are variants of one species group. An examination of the alleles of the two isolates named *N. perflava* (CCUG 32036 and CCUG 32112) that clustered with this group indicated that they were misidentified *N. mucosa* variants.

The non-human *N. macacae* isolate, CCUG 41451, is closely related to the other isolates in the *N. mucosa* group, whereas the other non-human isolates in this study, *N. canis* (CCUG 56775 T), *N. dentiae* (CCUG 53898) and *N. weaveri* (CCUG 4007 T), are only distantly related to the human isolates. The *N. macacae* type strain was isolated from the oropharnyx of a captive primate (Rhesus monkey), and the close sequence identity of isolates from primate hosts suggests that some *Neisseria* are likely to colonize more than one host species. The rod-shaped human isolates (*N. elongata* subsp. *glycolytica* ATCC 29315 and *N. bacilliformis* CCUG 50858 T) were not closely related to the other human isolates or to each other.

All of the isolates previously defined as *N. polysaccharea* were closely related to the *N. meningitidis*, *N. gonorrhoeae* and *N. lactamica* isolates, but did not represent a monophyletic group. Isolate 15883 was less closely related to the type strain ATCC 43768. This bacterium was isolated along with strain 25862 (CCUG 18031), which were the first examples of this species to be described in Germany (Berger, 1985). At the time of discovery, it was observed that both isolates were different from the type strain in that they did not grow on Thayer–Martin medium (TMM) and that isolate 15883 differed from the others in its degradation of sugar, but otherwise appeared identical to *N. polysaccharea*. Another study of *N. polysaccharea*, which included these isolates, found two distinct subsets among the isolates, with some resistant to colistin, an antibiotic used in TMM, and some susceptible, indicating further variability within this taxon and that the documentation of this species is incomplete (Anand *et al.*, 1991). Analysis of the set of genes employed here confirmed that isolate 15883 is distinct from other isolates of *N. polysaccharea* and could be either an *N. polysaccharea* variant or perhaps a separate species.

A subset of 98 genes (Table S3), which excluded the 53 ribosomal genes and consisted of 84 685 nt (amounting to 3.86 % of the query genome), were concatenated and used to reconstruct a neighbor-joining phylogeny. The same group structure as seen with the 246 gene analysis was evident (Fig. S2). Measures of *F_ST_* (Table 1), fixed differences and shared polymorphisms (Table 2) calculated on the basis of the species groups revealed in this report showed that with the exception of the *N. polysaccharea* isolates, there was high differentiation between species groups. *N. polysaccharea* was most closely related to *N. meningitidis*, with an *F_ST_* value of 0.35, the lowest number of fixed differences between species (511) and a high number of shared polymorphisms (2209).

**Table 1.** Gene flow between a set of 98 genes from seven species groups of *Neisseria* The divergent strains for which there is only one example (*N. mucosa* var. *heidelbergensis* CCUG 26878 T, *N. polysaccharea* 15883, *N. elongata* subsp*. glycolytica* ATCC 29315, *N. bacilliformis* CCUG 50858 T, *N. dentiae* CCUG 53898, *N. weaveri* CCUG 4007 T and *N. canis* CCUG 56775 T) have been excluded from this analysis. Figures above the diagonal are *F_ST_* values, those below are *P* values (significance level = 0.05). *Nmu*, *N. mucosa*; *Nsu*, *N. subflava*; *Npo*, *N. polysaccharea*; *Nme*, *N. meningitidis*; *Ngo*, *N. gonorrhoeae*; *Nla*, *N. lactamica*; *Nci*, *N. cinerea*. Numbers of isolates are shown in parentheses.

Species group	*Nmu*	*Nsu*	*Npo*	*Nme*	*Ngo*	*Nla*	*Nci*
*Nmu* (8)*		0.67	0.65	0.69	0.79	0.71	0.68
*Nsu* (13)†	0.00		0.67	0.71	0.78	0.72	0.68
*Npo* (5)	0.01	0.00		0.35	0.66	0.47	0.51
*Nme* (5)	0.00	0.00	0.01		0.71	0.59	0.64
*Ngo* (6)	0.00	0.00	0.00	0.00		0.81	0.80
*Nla* (5)	0.00	0.00	0.00	0.00	0.00		0.65
*Nci* (6)	0.00	0.00	0.00	0.00	0.00	0.00	

* This group includes *N. mucosa*, *N. sicca* and *N. macacae*.

† This group includes isolates defined as *N. subflava*, *N. perflava*, *N. flava* and *N. flavescens*.

**Table 2.** Fixed difference and shared polymorphisms between a set of 98 genes from seven species groups of *Neisseria* Figures above the diagonal are fixed differences, those below are shared polymorphisms. Numbers of isolates are shown in parentheses. *Nmu*, *N. mucosa*; *Nsu*, *N. subflava*; *Npo*, *N. polysaccharea*; *Nme*, *N. meningitidis*; *Ngo*, *N. gonorrhoeae*; *Nla*, *N. lactamica*; *Nci*, *N. cinerea*. The divergent strains for which there is only one example (*N. mucosa* var*. heidelbergensis* CCUG 26878 T, *N. polysaccharea* 15883, *N. elongata* subsp. *glycolytica* ATCC 29315, *N. bacilliformis* CCUG 50858 T, *N. dentiae* CCUG 53898, *N. weaveri* CCUG 4007 T and *N. canis* CCUG 56775 T) have been excluded from this analysis.

Species group	*Nmu*	*Nsu*	*Npo*	*Nme*	*Ngo*	*Nla*	*Nci*
*Nmu* (8)*		4493	4218	5015	6249	5169	4418
*Nsu* (13)†	2585		4907	5864	7112	5871	4893
*Npo* (5)	1642	2202		511	1738	1231	1237
*Nme* (5)	1227	1611	2209		1745	2065	2578
*Ngo* (6)	67	67	115	103		3439	4059
*Nla* (5)	1028	1383	1956	1321	82		2706
*Nci* (6)	1664	2303	2314	1555	96	1321	

* This group includes *N. mucosa*, *N. sicca* and *N. macacae*.

† This group includes isolates defined as *N. subflava*, *N. perflava*, *N. flava* and *N. flavescens*.

### Examination of ribosomal genes

The 53 *rps* genes represent ideal candidates for a bacterial classification scheme, as they are universally present, conserved and distributed around the bacterial chromosome. Concatenated gene sequences from the *rps* loci used in the rMLST scheme (Jolley *et al.*, 2012) have been shown to produce phylogenies that cluster species in groups substantiated by current nomenclature. The groups generated using rMLST data were also consistent and not dependent on the clustering algorithm used.

**Fig. 3.**
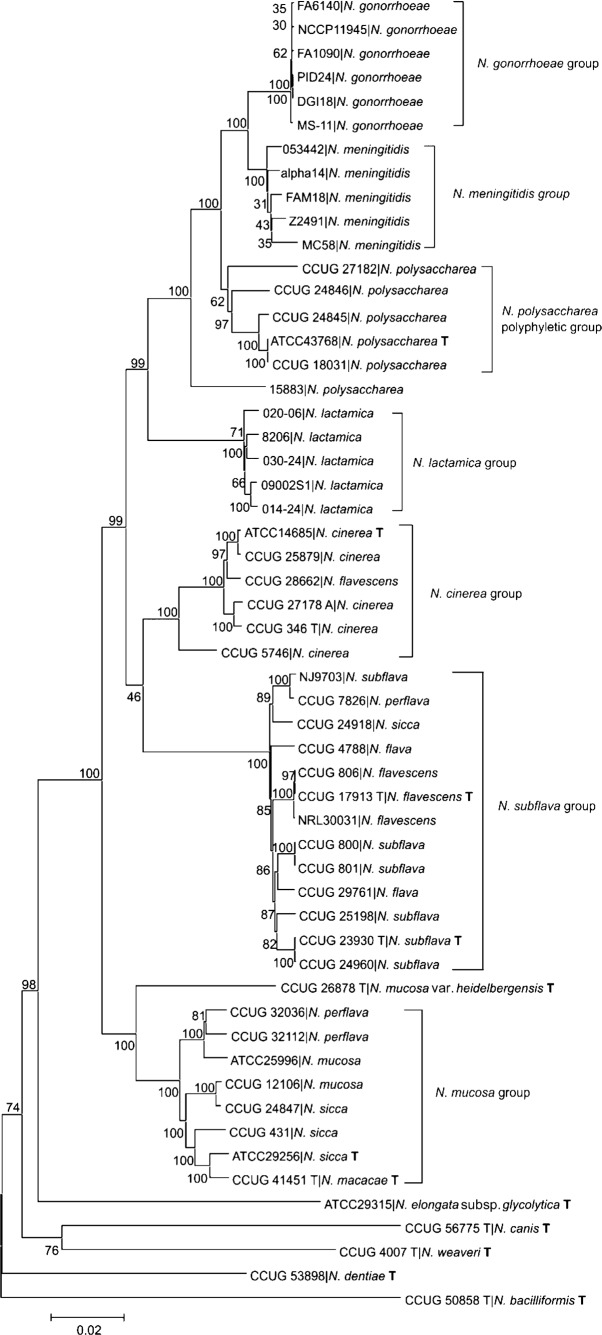
Evolutionary relationships among *Neisseria* based on concatenated sequences of 53 ribosomal protein genes. The evolutionary history was inferred using the neighbor-joining method. The percentages of replicate trees in which the associated taxa clustered together in the bootstrap test (500 replicates) are shown next to the branches. The analysis involved 55 nt sequences consisting of 21 398 nt. ‘T’ denotes type strain.

A *Neisseria* phylogeny was reconstructed from the concatenated *rps* gene sequences using the neighbor-joining method (Fig. 3), which showed the same groups as the phylogenies produced using either 246 or 98 concatenated gene sequence sets. These groups were also generated using neighbor-net (Fig. S3). The 53 genes used in these analyses consisted of 21 398 nt in total, amounting to 0.97 % of the genome of the reference meningococcal genome sequence FAM18.

Measures of gene flow, genetic differentiation and divergence among species supported the groups defined. An *F_ST_* value of 0.54 between *N. polysaccharea* and *N. meningitidis* (Table 3) indicated that *N. polysaccharea* is more closely related to *N. meningitidis* than the other species examined here. This is supported by the 239 fixed differences between *N. polysaccharea* and *N. meningitidis* (Table 4), which is the lowest number of fixed differences between species, and the number of shared polymorphisms, which is the highest number of shared polymorphisms between species (194). *F_ST_* values ranging from 0.74 (*N. gonorrhoeae* vs *N. polysaccharea*) to 0.95 (*N. lactamica* vs *N. gonorrhoeae*) indicate the higher level of differentiation between these other species. *N. gonorrhoeae* shared very few polymorphisms with other *Neisseria*, as *N. gonorrhoeae* isolates are likely to have descended from a single clone, have very low diversity and normally inhabit a different niche to other *Neisseria*. These data suggest that *N. meningitidis* and *N. gonorrhoeae* evolved from a common ancestor shared with isolates currently designated *N. polysaccharea*.

**Table 3.** Gene flow between 53 ribosomal genes from seven species groups of *Neisseria* Figures above the diagonal are *F_ST_* values, those below are *P* values (significance level = 0.05). *Nmu*, *N. mucosa*; *Nsu*, *N. subflava*; *Npo*, *N. polysaccharea*; *Nme*, *N. meningitidis*; *Ngo*, *N. gonorrhoeae*; *Nla*, *N. lactamica*; *Nci*, *N. cinerea*. Numbers of isolates are shown in parentheses. The divergent strains for which there is only one example (*N. mucosa* var. *heidelbergensis* CCUG 26878 T, *N. polysaccharea* 15883, *N. elongata* subsp. *glycolytica* ATCC 29315, *N. bacilliformis* CCUG 50858 T, *N. dentiae* CCUG 53898, *N. weaveri* CCUG 4007 T and *N. canis* CCUG 56775 T) have been excluded from this analysis.

Species group	*Nmu*	*Nsu*	*Npo*	*Nme*	*Ngo*	*Nla*	*Nci*
*Nmu* (8)*		0.79	0.76	0.80	0.85	0.80	0.75
*Nsu* (13)†	0.00		0.85	0.88	0.91	0.87	0.81
*Npo* (5)	0.00	0.00		0.54	0.74	0.82	0.78
*Nme* (5)	0.00	0.00	0.00		0.79	0.88	0.84
*Ngo* (6)	0.00	0.00	0.00	0.00		0.95	0.90
*Nla* (5)	0.00	0.00	0.01	0.01	0.01		0.82
*Nci* (6)	0.00	0.00	0.00	0.00	0.00	0.01	

* This group includes *N. mucosa*, *N. sicca* and *N. macacae*.

† This group includes isolates defined as *N. subflava*, *N. perflava*, *N. flava* and *N. flavescens*.

**Table 4.** Fixed difference and shared polymorphisms between 53 ribosomal genes from seven species groups of *Neisseria* Figures above the diagonal are fixed differences, those below are shared polymorphisms. *Nmu*, *N. mucosa*; *Nsu*, *N. subflava*; *Npo*, *N. polysaccharea*; *Nme*, *N. meningitidis*; *Ngo*, *N. gonorrhoeae*; *Nla*, *N. lactamica*; *Nci*, *N. cinerea*. Numbers of isolates are shown in parentheses. The divergent strains for which there is only one example (*N. mucosa* var. *heidelbergensis* CCUG 26878 T, *N. polysaccharea* 15883, *N. elongata* subsp. *glycolytica* ATCC 29315, *N. bacilliformis* CCUG 50858 T, *N. dentiae* CCUG 53898, *N. weaveri* CCUG 4007 T and *N. canis* CCUG 56775 T) have been excluded from this analysis.

Species group	*Nmu*	*Nsu*	*Npo*	*Nme*	*Ngo*	*Nla*	*Nci*
*Nmu* (8)*		916	1147	1259	1431	1162	834
*Nsu* (13)†	162		1247	1450	1677	1298	863
*Npo* (5)	133	142		239	394	1075	950
*Nme* (5)	71	66	194		314	1103	1047
*Ngo* (6)	12	4	11	3		1307	1240
*Nla* (5)	48	46	115	57	5		829
*Nci* (6)	162	136	187	84	3	80	

* This group includes *N. mucosa*, *N. sicca* and *N. macacae.*

† This group includes isolates defined as *N. subflava*, *N. perflava*, *N. flava* and *N. flavescens*.

An analysis of the individual ribosomal allele sequences used for rMLST showed that some identical allele sequences were shared among different species groups, consistent with a common ancestry for these genes that encode proteins that are under stabilizing selection for functional conservation. Another explanation is that there is frequent genetic recombination between species, with recombination acting as a mechanism for repairing core genes rather than as a method of diversification (Treangen *et al.*, 2008). Identical ribosomal alleles shared between species were more frequent between *N. polysaccharea* and other *Neisseria* than between any other species group and the rest of the genus examined here. This suggests that if recombination is frequent among the ribosomal genes of *Neisseria*, then carriage of *N. polysaccharea* is more common than currently recognized.

In contrast, there was little support for frequent inter-species recombination among the MLST alleles identified using BIGSdb, as these were unique to each species group defined here. This was also true when the whole genes from which the MLST fragments were extracted were examined (data not shown). This indicates that metabolic housekeeping genes evolve and diverge as they adapt to a particular niche, and as they diverge, distinct alleles become evident that are specific to a particular species, consistent with previous findings (Bennett *et al.*, 2010).

Further analyses of the individual ribosomal allele sequences for these isolates provided further evidence for isolate or species misclassification. For example, the *N. flavescens* isolate CCUG 28662, which grouped with *N. cinerea*, had 19 ribosomal gene sequences identical to other *N. cinerea* isolates, including 13 identical to the type strain, but none identical to any other *N. flavescens* isolate examined, confirming that it is *N. cinerea*. It was isolated in Sweden in 1991, and is not closely related to the *N. flavescens* isolates originally identified during an outbreak of meningitis in Chicago in 1928 (Branham, 1930).

All the isolates within the *N. mucosa* group shared similar ribosomal gene sequences and can be considered variants of one species, as is the case for the *N. subflava* variants. The close relationship between *N. macacae* and other *Neisseria* within the *N. mucosa* group was supported by the observation that 13 *N. macacae* ribosomal gene sequences were identical to sequences present in other *N. mucosa* group genomes. Also in the *N. mucosa* group were two isolates identified originally as *N. subflava* biovar *N. perflava* (CCUG 32112 and CCUG 32036). These shared no ribosomal alleles with other isolates in the *N. subflava* group, but shared a large number with isolates clustering in the *N. mucosa* group, confirming their identity as *N. mucosa* variants. Another isolate, which was originally identified as *N. sicca* (CCUG 24918), but clustered with the *N. subflava* group, shared ribosomal alleles with all subspecies from the *N. subflava* group, including *N. flavescens*, but none with any isolates clustering in the *N. mucosa* group, confirming that it is an *N. subflava* variant, along with the *N. flavescens* isolates in this group.

### *Neisseria* classification

The availability of genomic data and the development of the BIGSdb platform have facilitated a classification method (rMLST) which has sufficient power to classify species within the genus *Neisseria* rapidly and reliably. Species assignments for the human isolates *N. meningitidis*, *N. gonorrhoeae* and *N. lactamica* are well established, but a number of other species require some reclassification. These data indicate that the species *N. sicca* and *N. macacae* should be classed as variants of *N. mucosa*, and that *N. mucosa* var. *heidelbergensis* is sufficiently diverse to be assigned species status (*Neisseria heidelbergensis*). *N. flavescens* is closely related genetically to the *N. subflava* variants and could be considered a variant of this species (*Neisseria subflava* var. *flavescens*). *The N. cinerea* isolates formed a distinct group, although isolate CCUG 5746 was more dissimilar to the other representatives of this species. This isolate may form a distinct variant of this species, but more data from other *N. cinerea* isolates would be required to confirm this.

The isolates currently designated *N. polysaccharea* examined here form a polyphyletic group. Data from historical studies indicate that *N. polysaccharea* is diverse (Anand *et al.*, 1991), is more closely related to *N. meningitidis* than other *Neisseria* species (Guibourdenche *et al.*, 1986; Zhu *et al.*, 2003), is carried by children of primary school age (Cann & Rogers, 1989; Saez-Nieto *et al.*, 1985) and may act as a reservoir for antibiotic resistance (Saez-Nieto *et al.*, 1990). Taken together these data indicate that further examination of the *N. polysaccharea* variants is required to define this species group accurately, and that additional research is needed to determine its genetic relationship to *N. meningitidis*, its epidemiology and its rate of carriage in young children. The most diverse of the *N. polysaccharea* isolates (15883) requires reclassification, as it differs from the other *N. polysaccharea* variants both phenotypically (Berger, 1985) and genotypically, and is less closely related to *N. meningitidis* than the other isolates designated *N. polysaccharea*. A suggested name for the reclassification of this strain is *Neisseria bergeri*.

### Conclusions

Reliable identification and classification of bacteria is important in all areas of microbiology, but is essential in clinical applications. It is important that commensal *Neisseria* are accurately distinguished, as some may be misidentified as pathogenic species, and occasionally some are isolated from unusual sites and must be correctly identified for clinical purposes (Knapp, 1988). Accurately identified bacterial species are an essential starting point to investigate the genetic determination of phenotypes by the comparison of related isolates that exhibit diverse properties. The availability of whole-genome sequences has greatly increased the number of possible comparative studies, but it is essential that the isolates used in such investigations are well characterized to realize the opportunities presented by association studies of diverse phenotypes with particular genotypes. As in many micro-organisms, the accessory genome is widely shared among *Neisseria* that have distinct pathologies; for example, many ‘virulence-associated’ genes identified for *N. meningitidis* and *N. gonorrhoea* are also present in the non-pathogen *N. lactamica*. Consequently, it is necessary to examine sequence divergence in core genes to accurately characterize bacterial isolates. This analysis demonstrates that in the genus *Neisseria*, reproducible species groups can be generated from various sets of genes including a ‘minimal core genome’, the 53 *rps* genes. These groups are largely congruent with previous nomenclatures, and therefore this approach represents an effective and rapid method for taxonomic classification that can be readily applied to other bacterial groups. The method has the potential to replace approaches such as DNA association studies as a reproducible and generally applicable basis for bacterial identification and classification.
